# Stress during the first 1,000 days of life in humans, when everything begins

**DOI:** 10.3389/fnhum.2026.1781545

**Published:** 2026-05-12

**Authors:** Tania Vitalis, Catherine Verney

**Affiliations:** Université Paris Cité, Inserm U1141, NeuroDiderot, Paris, France

**Keywords:** adaptive, anatomy, cognition, coping, development, physiology, stress, traumatic

## Abstract

Brain development from fetal life to early childhood occurs in highly sensitive periods, during which stress exposure—adaptive or toxic, prenatal or postnatal—can shape neural circuits involved in emotional regulation, particularly amygdala-centered networks. This review synthesizes current evidence on the biological mechanisms of early-life stress and protective interventions. It is based on a narrative review integrating original research, review articles, and international guidelines selected for relevance. Severe or prolonged early-life stress, including prenatal adversity, maternal anxiety, or environmental challenges, can disrupt body–brain interactions, alter hypothalamic–pituitary–adrenal axis activity, reduce vagal tone, and impair connectivity in the amygdala, hippocampus, and salience network. Epigenetic modifications in genes link early adversity to altered stress reactivity and emotional regulation. Functional MRI and physiological assessments indicate these changes are detectable before birth and during the first 1,000 days of life. Supportive caregiver interactions—through polyvagal-mediated synchrony, attachment, and social engagement—promote physiological regulation and healthy neural development. Interventions such as skin-to-skin contact reduce cortisol levels, enhance vagal activity, and increase oxytocin release. Traumatic early life stress can profoundly influence neural, hormonal, and epigenetic pathways, but positive caregiving and interventions can foster resilience and optimize neurodevelopment. These findings highlight the critical need to monitor and support stress regulation during the first 1,000 days of life.

## 1 Introduction

Healthy child development is a central concern in public health. A growing body of research supports the theory of the Developmental Origins of Health and Disease (DOHaD), which highlights how early environmental exposures influence physical, psychological, and behavioral outcomes across the lifespan (Hoffman et al., 2021; Lapehn and Paquette, 2022; Nobile et al., 2022). Among these factors, early-life stress plays a particularly critical role. Its biological effects are partly mediated by epigenetic modifications, especially during pre- and postnatal development. Increasing attention has been given to the first 1,000 days of life—from conception to 2 years of age—as a sensitive period for brain development and the prevention of long-term health inequalities (New Lancet Series, 2017; Global Wellness Institute, 2022; Early development, UNICEF, 2023). In this context, non-governmental organizations such as UNICEF and the Global Wellness Institute have promoted initiatives aimed at supporting both families and early childhood professionals. Notably, individuals exposed to high levels of early stress are at increased risk of developing difficulties in emotional regulation (Roelofs, 2017). The Harvard University’s Center on the Developing Child distinguishes three types of stress observable across development: positive stress, which supports adaptive functioning; tolerable stress, which can be buffered by supportive relationships; and toxic stress, which is severe or prolonged and can have lasting negative effects on health, behavior, and neurodevelopment (National Scientific Council, 2014; Shonkoff and Phillips, 2000). In this context, the timing of the development of the anatomical systems involved in stress processes - from in utero life through early childhood - helps shape how adaptive and toxic stress are perceived and managed. Sensitive and critical periods play a key role in the proper maturation of sensorimotor, behavioral, and cognitive functions (Box 1). Early before birth, biological systems underlying stress responses are activated (Canini et al., 2023), although the underlying mechanisms are only partially understood. Around birth, the quality of an infant’s interactions with their sensory and social environment influences the development of brain networks referred here as the connectome, in particular the vagal system associated with attachment processes. When stress reaches a toxic or traumatic intensity, it may lead to harmful biological, psychological, and social outcomes. But the infant brain is highly plastic in early time periods and the effects of environmental stressors may be reversible. Early identification and intervention are therefore essential to promote resilience and reduce long-term vulnerability. Given the heightened vulnerability of early life, it is crucial to define optimal care strategies not only for the child, but also for parents, caregivers, and parent–child dyads.

Box 1 **Human brain development.**

**Figure 1:**
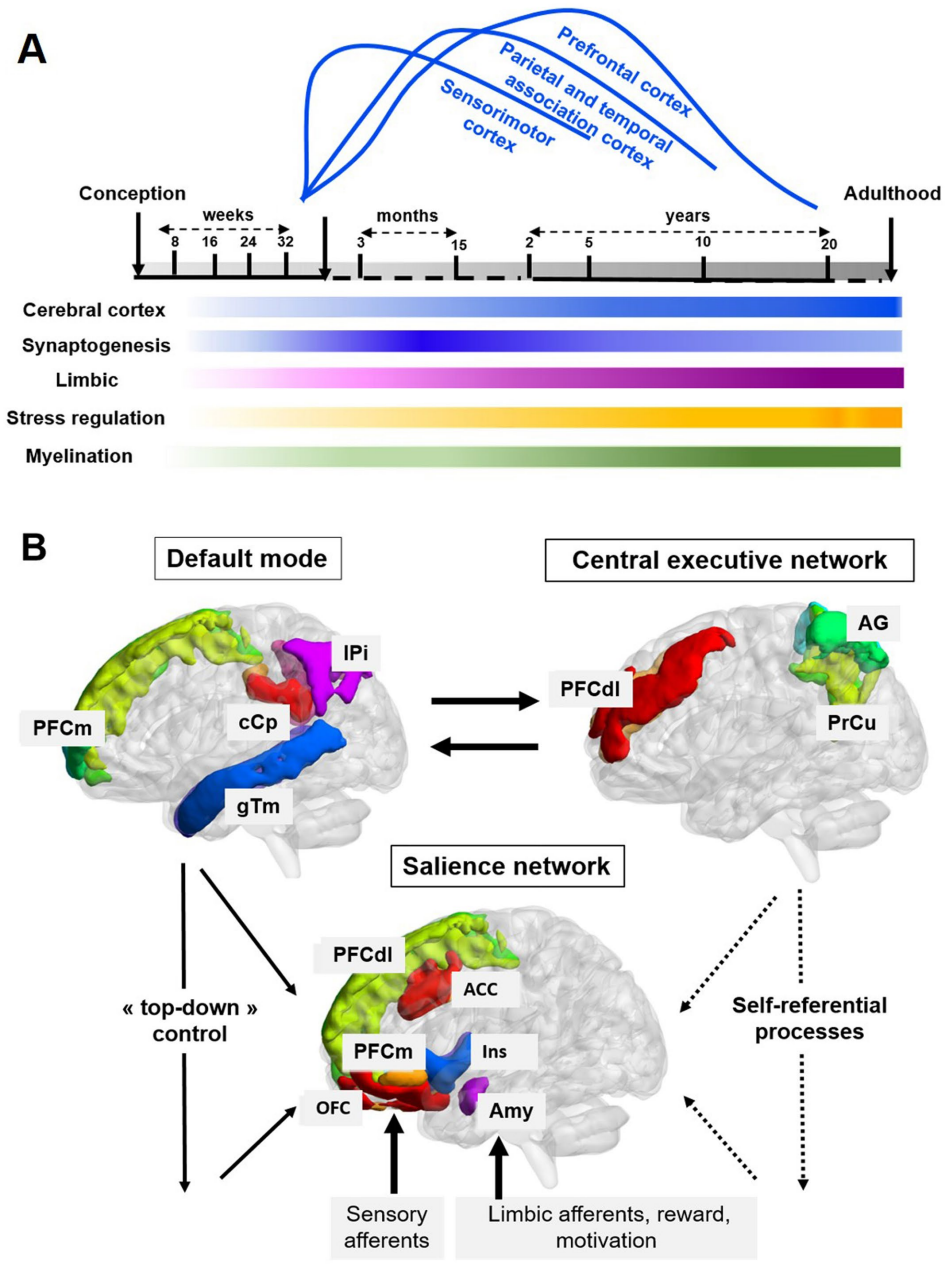
**(A)** Developmental sequences of brain systems related to stress. The intensity of each color reflects the degree of maturation/function of each system. Overall, cortical connections (azure blue) and cortical synaptogenesis (cobalt blue) begin around 18 gestational weeks (GW) and increase, peaking in sensorimotor systems around birth. These events occur later, around 15 months postnatal, for the parietal and temporal associative cortices, followed by the prefrontal cortex later in development. Subcortical limbic nuclei, including the thalamus, amygdala, and hippocampus, are shown in pink. Structures involved in stress regulation (autonomic nervous system and corticotropic HPA axis appear in yellow). Myelination cycles (green) of different connectome networks begin in the third trimester and continue until around 20 years of age [adapted from Bourgeois (1997), Huttenlocher and Dabholkar (1997), and Lammertink et al. (2021)]. **(B)** The three main cerebral hubs/networks active around birth (Box 1) (Bressler and Menon, 2010). The default mode network (DMN) is active during sleep and quiet wakefulness (interoception) and deactivates during external stimulus processing by the executive function network, which requires attention directed toward task execution (dorsolateral prefrontal cortex – dlPFC, angular gyrus – AG, precuneus – PrCu). This executive network strengthens sequentially with learning. At birth, two networks are strongly interconnected: (1) the DMN, including the medial prefrontal cortex (mPFC), posterior cingulate cortex (PCC), inferior parietal lobule (IPL), and middle temporal gyrus (MTG); and (2) the salience network, including the medial prefrontal cortex (mPFC), orbito-frontal cortex (OFC), anterior cingulate cortex (ACC), insula (Ins), and amygdala (Amy). The salience network is engaged in perceiving and integrating emotional and sensory stimuli. It mediates the transition between the DMN (“top-down” control) and executive functions (self-referential processes) necessary for adaptive behavior [adapted from Nelson and Gabard-Durnam (2020), van den Heuvel and Thomason (2016) and Box 1].

Brain morphogenesis is the expression of genetic programming, a series of genetic, molecular, and cellular events that are induced and/or inhibited in strict spatiotemporal sequences. During the embryonic period [up to 8 weeks of gestation (WG) in humans], neurogenesis of the subcortical brain regions (brainstem, midbrain, diencephalon (hypothalamus, thalamus), amygdala) is largely completed. Most neurons of the cerebral cortex are generated between 7 and 16 WG. The sensorimotor and associative cortical areas are activated in the perception-action processing around birth. The pyramidal motor pathway is present from the second trimester of gestation in humans, while non-pyramidal motor regulations mature with walking around 1 year of age until the mastery of writing around 7–8 years of age. Touch, taste, and proprioception are present around the third month of pregnancy, hearing around the sixth month, while vision is activated at birth. At the end of gestation and around the time of birth, programmed morphogenesis is complete. The encounter between the fetus and the newborn and their environment stimulates and stabilizes the synapses and axonal networks that will later form the basis of everyone’s unique connectome. Sensory, motor, and emotional experiences select functional synapses and axonal arborizations, eliminate and prune unstimulated axons and synapses (Huttenlocher and Dabholkar, 1997; Bourgeois, 1997). These sequences of progressive stabilization of different networks/hubs correspond to sensitive or critical periods. Vegetative, sensorimotor, parietal, temporal, and then prefrontal associative axonal networks are progressively structured until adulthood (Figure 1A). The quality of the environment during these sensitive periods of connectome shaping specific to each individual is essential for its proper development (Nelson and Gabard-Durnam, 2020). The human connectome (the complete map of neural connections) involves three main networks/hubs, the default network, the executive control network and the salience network (Bressler and Menon, 2010) (Figure 1B). In default mode, the brain is not engaged in any task requiring attention or the processing of an external stimulus (in a state of calm wakefulness or sleep), unlike executive control, which requires the mobilization of attention and working memory in adapting behaviors toward a given task. The unique salience network for everyone is activated in the perception and integration of sensory and emotional stimuli by mobilizing limbic networks, including the dopaminergic reward circuit (pleasure/motivation) (Lammertink et al., 2021). The salience network would allow the transition from a “default mode” to the executive control necessary for adapting behaviors.

This review aims to synthesize current evidence on the biological mechanisms of early-life stress and protective interventions and is based on a narrative review integrating original research articles, review papers, and international guidelines, selected for their relevance to the topic.

## 2 Ontogeny of stress-responsive anatomical structures

The various brain systems that manage stress (Herman et al., 2016; Mc Evan, 2007), such as the autonomic nervous system, the corticotropic (hypothalamic–pituitary–adrenal) HPA axis, monoaminergic networks, and cortico-limbic circuits, begin to form during the prenatal period following developmental sequences outlined in Figure 1A and Box 1 (Lammertink et al., 2021; Nelson and Gabard-Durnam, 2020).

The HPA axis develops during the embryonic period (up to the 8th GW), with differentiation extending into the third trimester of gestation (Lammertink et al., 2021). However, the functional role of cortisol in the fetus remains not clearly defined as it is difficult to distinguish fetal from maternal contribution (Davis et al., 2017).

The neurogenesis of monoaminergic brain nuclei (noradrenaline/adrenaline, dopamine and serotonin) occurs between the 4th and 7th GW followed by the ascending and/or descending axonal projections. During the fetal period noradrenergic and dopaminergic axons invade the cerebral cortex in an adult-like cortical distribution by the end of the second gestational trimester (Sundström et al., 1993; Verney, 1999; Verney et al., 2002). The different nuclei of the amygdala complex become identifiable around the 15th GW and are differentiated at birth (Lammertink et al., 2021). The thalamus, which is closely connected to the amygdala, serves as a crucial sensory and limbic relay in cortical activation triggered by stress. The different sensory, limbic, motor, and cognitive thalamic nuclei become identifiable around the 26th GW. Thalamo-cortical connections set in around the 17th GW and extend until birth (Bourgeois, 1997; Lammertink et al., 2021). The development of cortical connections follows a medio-lateral and postero-anterior gradient (van den Heuvel and Thomason, 2016). The connectome (the complete map of neural connections) of midline areas, such as the anterior and posterior cingulate cortex and the medial frontal areas (orbito-frontal and ventro-medial), are part of early proto-networks like the salience network and the default mode network (Figure 1B). Imaging data suggest these proto-networks are active around the time of birth, long before the executive function (dorsolateral prefrontal cortex) and attention (parieto-frontal networks) systems are mature (Lammertink et al., 2022; Menon, 2011; Ouyang et al., 2019). At birth, language acquisition networks already resemble their adult configuration, as do the fusiform gyrus connections, responsible for face recognition (Dubois et al., 2016; van den Heuvel et al., 2018).

## 3 Development of body–brain stress circuits

**Figure 2:**
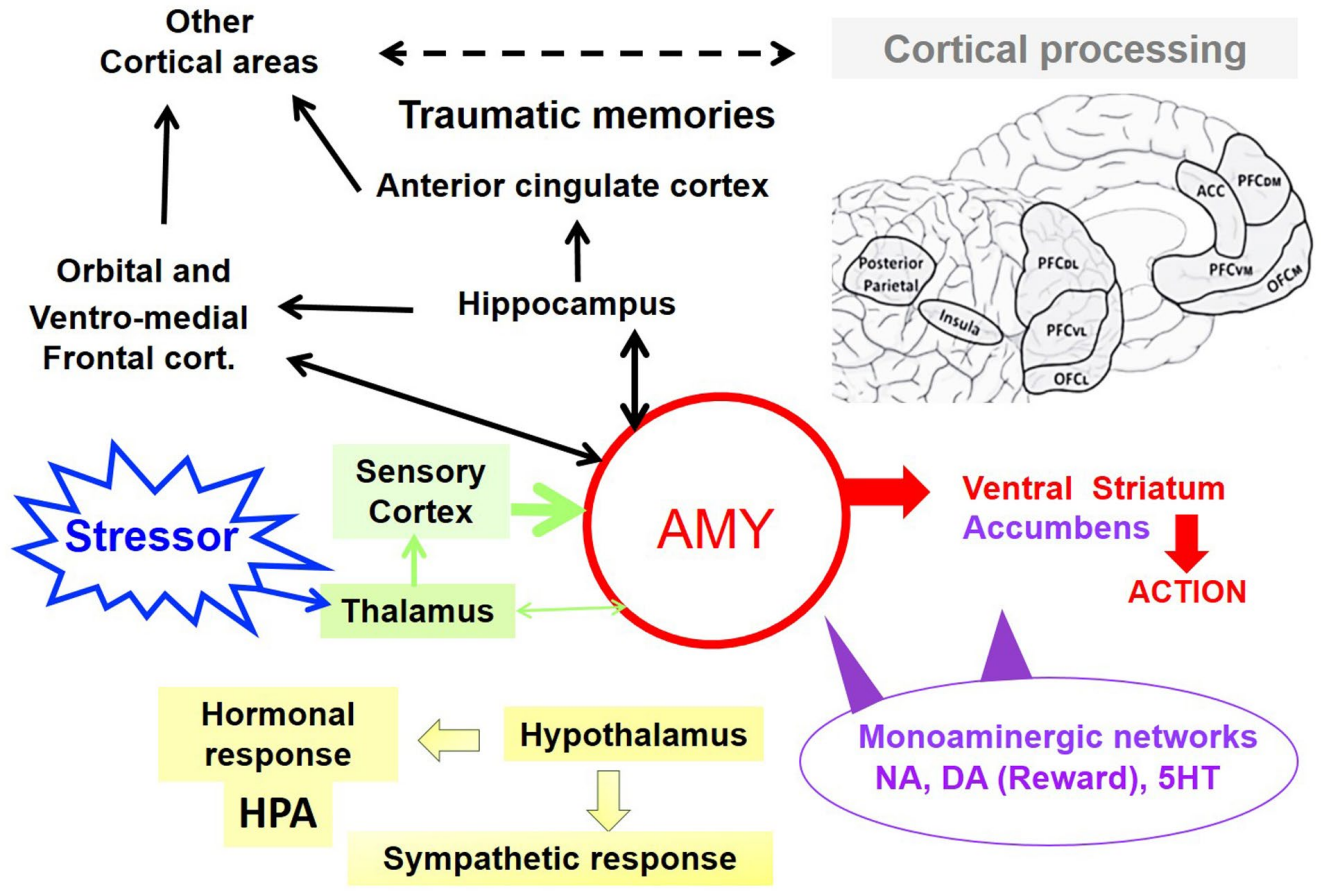
Hub of the amygdaloid complex and limbic structures. Input pathways include sensory stimuli (pale green) and autonomic signals (hypothalamus) that activate the corticotropic HPA axis (physiological response; yellow). Contextual information processed by memory networks (hippocampus) and cortical limbic regulation is transmitted to the orbital and ventro-medial frontal cortex. Via the cerebral processing, traumatic memories may anchor the anterior cingulate cortex. The nucleus accumbens and ventral striatum regulate motivation and action selection within a basal ganglia loop (red). Neuromodulatory networks (violet), noradrenaline (NA), dopamine (DA), serotonin/5-hydroxytryptamine (5-HT) are distributed across multiple brain nuclei, including the amygdala, hippocampus, and cortical areas.

A sudden event (stressor) activates the sensory organs, which—except for vision—are functional before birth, and transmit information very rapidly (within tens of milliseconds) to the sensory cortical areas and the amygdala (Figure 2) (Mc Evan, 2007). From the last trimester on, functional synchronization between fetal limbic regions, including the amygdala and the neocortex has been demonstrated using resting-state fMRI, indicating the presence of limbic functional connectivity in utero (Dubois et al., 2016; Canini et al., 2023).

In infants, the amygdala-centered network underlies early processing of emotional salience and basic valence, whereas frontal regulatory circuits remain largely immature (Leppänen and Nelson, 2009; Gee et al., 2013). By early childhood (~3 years), the amygdala–hippocampal network supports the basic evaluation of emotional experiences and valence. Meanwhile, frontal regulatory mechanisms are still immature, limiting flexible and context-dependent regulation (Gee et al., 2013; Tottenham, 2014). Later in childhood, the gradual maturation of parieto-frontal and frontal circuits supports increasing flexibility in contextual awareness and the more refined evaluation of emotional situations, enabling more adaptive action selection (Gee et al., 2013; Tottenham, 2014).

**Figure 3:**
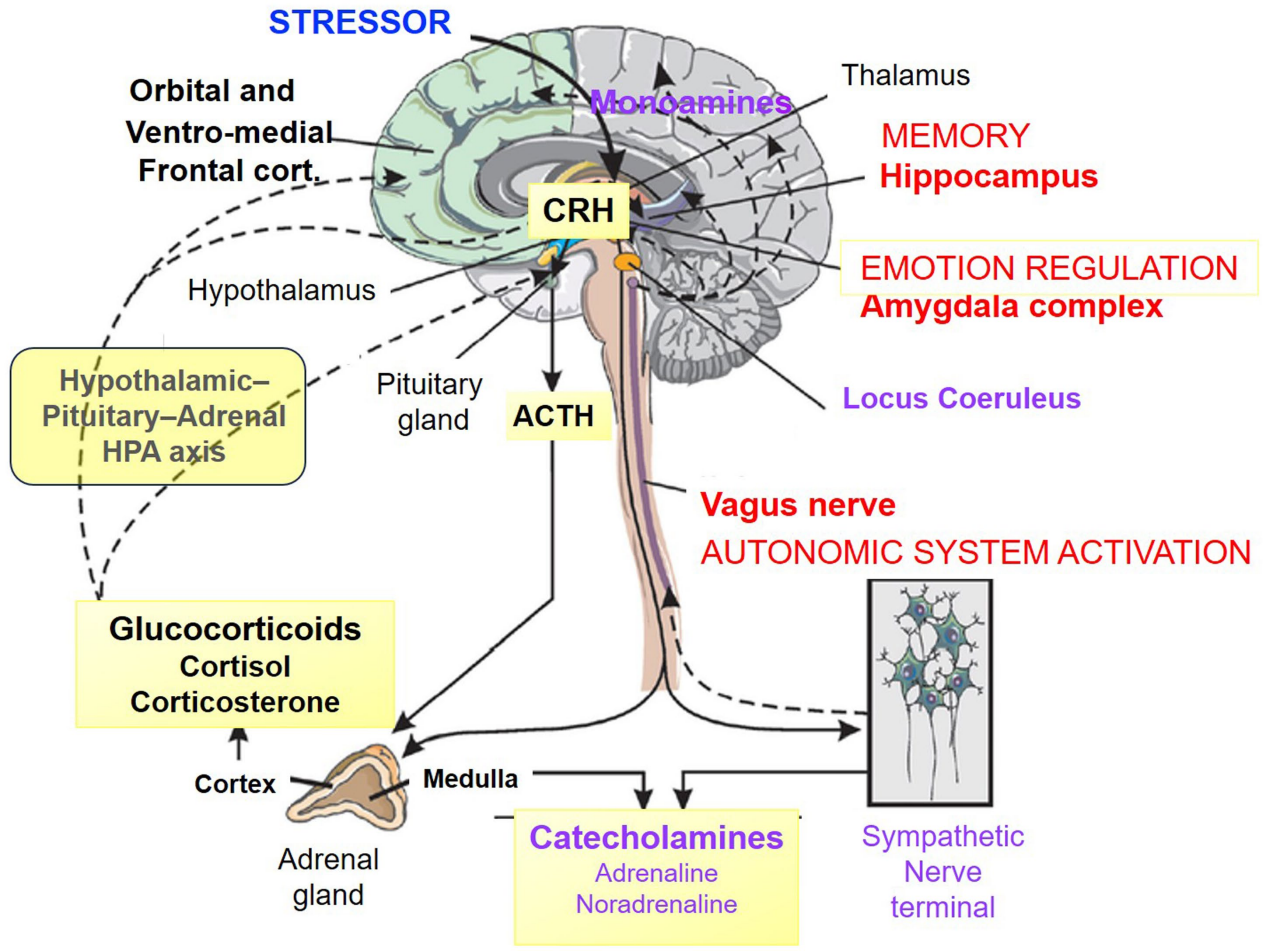
Biological body–brain responses to acute stress with activation of the corticotropic axis (in yellow). In response to stress, hypothalamic neurons release corticotropin-releasing hormone (CRH), which stimulates adrenocorticotropic hormone (ACTH) secretion from the anterior pituitary. ACTH promotes cortisol release from the adrenal cortex, which activates glucocorticoid receptors widely expressed in the brain, including the amygdala, developing hippocampus, and frontal cortex. Cortisol initially enhances alertness and situation evaluation and is regulated via negative feedback on the hypothalamus and pituitary. Under chronic or repeated stress, this regulation may become dysregulated. Simultaneously, CRH interacts with the locus coeruleus to increase noradrenergic activity through the sympathetic nervous system. Adapted from Figure 1 in Verney et al. (2021).

The HPA axis coordinates the organism’s physiological response through a tightly regulated neuroendocrine cascade (Figure 3). Stress triggers activation of hypothalamic neurons that release corticotropin-releasing hormone (CRH), which stimulates the secretion of adrenocorticotropic hormone (ACTH) from the anterior pituitary. ACTH subsequently promotes the release of cortisol from the adrenal cortex into the bloodstream. Cortisol binds to and activates glucocorticoid receptors, which are widely expressed throughout the brain, particularly within components of the HPA axis and in key limbic structures, including the amygdala, the developing hippocampus, and the frontal cortex (Herman et al., 2016) (Figure 3). The action of cortisol initially promotes alertness and supports the evaluation of the situation. This response is regulated by a negative feedback mechanism, whereby cortisol inhibits both the hypothalamus and the pituitary. The HPA axis typically returns to basal levels following adaptive stress responses but may remain dysregulated under conditions of chronic or repeated stress. In parallel, CRH interacts with the locus coeruleus to promote noradrenergic activity via the sympathetic nervous system.

Adaptive responses to stress are also supported by dopaminergic reward related circuits, extensively characterized in animal studies and conceptual frameworks of emotional regulation (LeDoux, 2012; LeDoux and Pine, 2016). In humans, as mentioned above, the mesocorticolimbic dopaminergic system is in place before birth (Verney, 1999; Verney et al., 2002). Furthermore, behavioral evidence indicates that circuits mediating basic reinforcement, such as responses to sweet taste, are already functional in infants [Beauchamp and Moran, 1982; reviewed in Berridge (2000)]. In older children, task based on functional MRI and developmental meta-analytic studies show robust activation of reward processing networks, with age dependent patterns particularly in striatal and insular regions (Yaple et al., 2020).

## 4 Critical periods and the impact of stress on the connectome

During brain development, the sensory, motor, and emotional experiences of the newborn select functional synapses and axonal arborizations, sculpting and stabilizing the unique connectome, the complete map of neural connections in a brain for everyone (information in Box 1). These sequences of gradual stabilization correspond to sensitive or critical periods in everyone’s development (Kaiser et al., 2018).

### 4.1 Modification of global network activation

Previously, we schematically defined the three main networks that characterize the human connectome: the default mode network, the executive control network, and the salience network, which is involved in emotion regulation and stress responses (Bressler and Menon, 2010; Figure 1B). Chronic stress in extremely premature infants leads to hyperactivation of the proto-salience network at the expense of the default mode network (Ross et al., 2019), which may increase the risk of long-term emotional, cognitive, and social difficulties (Montagna and Nosarti, 2016). Neural plasticity provides real potential for recovery (Shonkoff and Phillips, 2000).

### 4.2 Impact on the amygdala complex

As described above, during early development, the amygdala acts as the central hub for stress processing, coordinating physiological and behavioral responses. Chronic or intense stress exposure from birth on can induce hyperactivation of amygdala circuits, disrupting the balance between excitatory glutamatergic signaling and inhibitory GABAergic modulation. This prolonged excitation, coupled with impaired inhibition, can drive hypertrophy and structural remodeling, resulting in a later increase in amygdala volume (Zhang et al., 2019; Roozendaal et al., 2009) (Figures 1B) (Box 1).

Functional feedback loops of the stress circuitry between the amygdala, medial frontal cortex, insula, and anterior cingulate cortex (ACC) emerge from birth on (Hartley and Phelps, 2010; Rogers et al., 2017; Young et al., 2018; Zhai et al., 2019; Fermin et al., 2023) (Figure 2). The insula, which integrates visceral interoceptive awareness, is over-solicited during stress, while the ACC, which is involved in decision-making, emotional regulation, and the processing of traumatic memories, is particularly engaged in stress responses. Toxic stress activates these loops exacerbating this excitation/inhibition imbalance and potentially interfering with long-term limbic and frontal regulation (Brady et al., 2022; Humphreys et al., 2020) (Box 2).

Box 2 **The multiple functions of the vagus nerve—the polyvagal theory [based on Porges (2011), Porges (2022), and van der Kolk (2014)].**

Around birth, vagal tone maintains the physiological homeostasis of various organs, particularly cardiorespiratory rhythms. It acts as a brake by inhibiting sympathetic activation, thus maintaining a low heart rate and adjusting blood flow to metabolism. The dorsal motor nucleus of the vagus nerve, which is autonomic and originates unmyelinated nerve endings, primarily regulates fine cardiac adaptations. In parallel, the nucleus ambiguus, the ventral source of the myelinated vagus nerve, ensures rapid cardiac and metabolic adaptation under all circumstances. The nucleus ambiguus has multiple connections with different nuclei of the cranial nerves controlling the muscles of the face and head (facial expressions, vocalizations, hearing, vision), the amygdala, the hypothalamus (corticotropic axis), and the temporal and parietal cortical areas. These connections create a synergy between visceral states and spontaneous social behaviors through feedback pathways. The myelinated vagal system begins to function around the third trimester of gestation, progressively integrating mature cortical regulations during the first year of life. The ventral polyvagal system activates the infant’s facial sensorimotor pathways associated with neuroception (non-conscious detection of safety or danger by the nervous system). It allows the detection of cues indicating whether the environment is dangerous or not, whether people are trustworthy, which deactivates defense mechanisms and contributes to social engagement and attachment (Porges, 2022; Busuito et al., 2019).

These disruptions facilitate the consolidation of implicit, unconscious fear memories before hippocampal-dependent explicit memory becomes fully functional around 2–3 years of age (Gee et al., 2013; Tottenham, 2014) (Figure 3). Repeated adversity strengthens these circuits through morphological and epigenetic modifications, which may hinder plasticity and resilience during sensitive developmental windows (see Box 1).

## 5 High intensity of stress can induce epigenetic modifications

**Figure 4:**
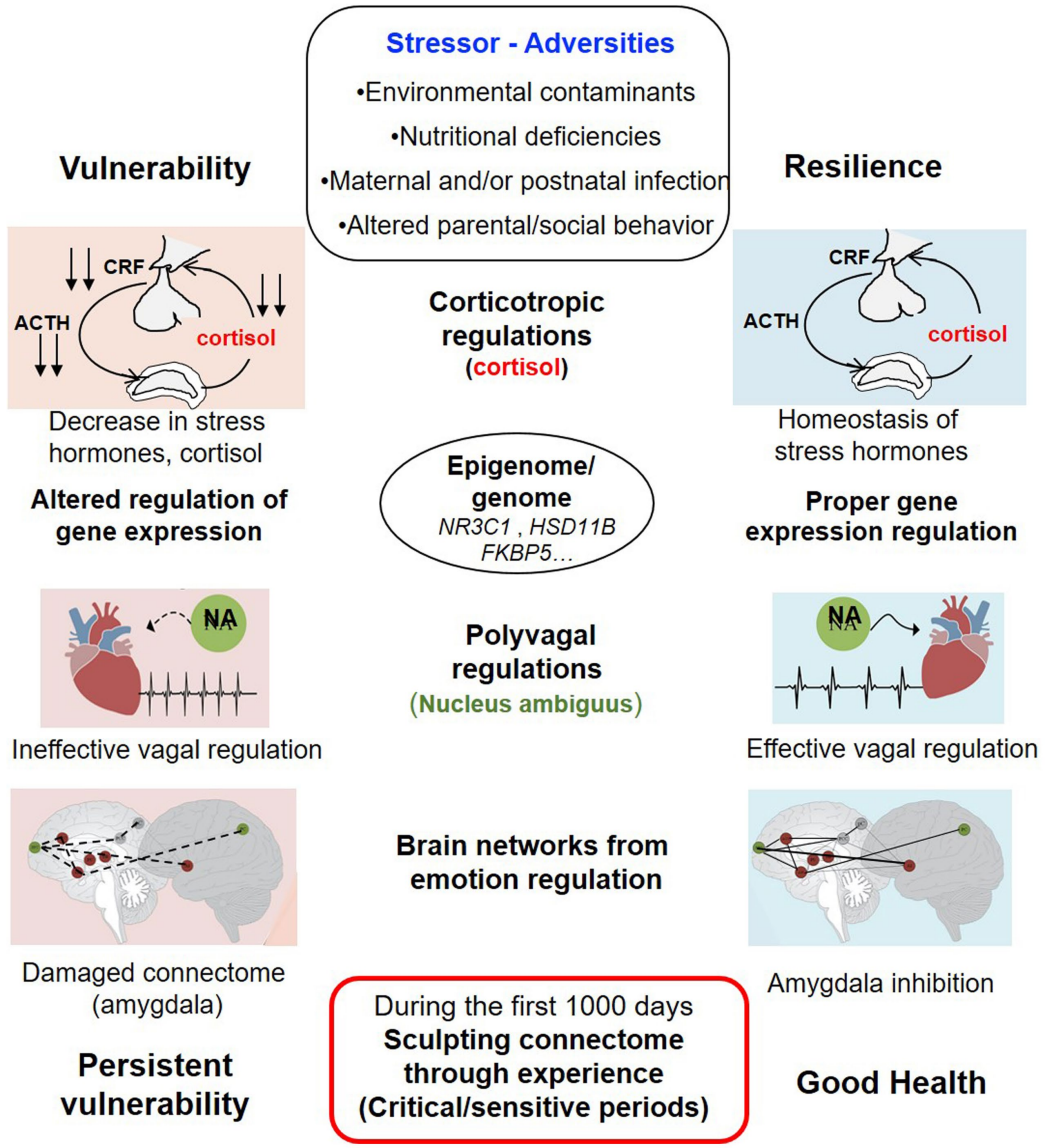
In preterm and/or term neonates exposed to various adversities or chronic stress, the different biological regulations leading to resilience are those: 1/ of the corticotropic axis (hypothalamo–pituitary–adrenal) leading to homeostasis of stress hormones (ACTH, adrenocorticotropic hormone; CRF, corticotropin-releasing factor) and cortisol; 2/ of the ventral polyvagal system (NA, nucleus ambiguus); 3/ of the regulation of the expression of specific genes: NR3C1, HSD11B, FKBP5 (epigenome); 4/ of cerebral networks for emotion management, particularly those inducing amygdala inhibition (dark red: limbic cortex, green: associative cortex). Dysregulation of these systems during critical developmental periods (vegetative, sensory, sensorimotor, limbic) can affect brain homeostasis, influencing trajectories toward resilience or subsequent vulnerability. Adapted from Figure 2 in Verney and Vitalis (2023).

Environmental adversity, including perinatal stress in its many forms, can induce epigenetic modifications in the mother (particularly at the placental level), fetus and newborn, affecting the regulation of the corticotropic axis (cortisol), inflammation, monoaminergic systems, neuropeptides, and the gut microbiome (Figure 4) (Cameron and Schoenfeld, 2018). Mechanisms involved may include changes in DNA methylation, histone acetylation or phosphorylation, or non-coding RNAs (Nobile et al., 2022). In healthy adults, stress exposure triggers expression of a set of genes coding for inflammatory mediators, known as the Conserved Transcriptional Response to Adversity response (Ross et al., 2019). During the third trimester of pregnancy, exposure to intense stress increases the release of pro-inflammatory cytokines such as IL-1, IL-6, TNF-α, and C-reactive protein (CRP)—potentially leading to premature birth. In mothers, stress also increases circulating cortisol. However, much of this cortisol is converted to cortisone by the placenta, via the enzyme HSD11B2 (11β-hydroxysteroid dehydrogenase type 2). At the same time, methylation of the *HSD11B* gene (which codes for this enzyme), and of *NR3C1* (which encodes the glucocorticoid receptor) increases which reduce their expression (Capron et al., 2018). The resulting high cortisol levels affect gene expression, including that of *FKBP5*, which encodes a protein that reduces the binding affinity of cortisol to its receptor. Maternal stress during pregnancy has been linked to increased *FKBP5* methylation in newborns, reducing their ability to bind cortisol and predisposing them to agitation at birth (Monk et al., 2016). Gestational stress also disrupts circulating monoamines. High levels of adrenaline and noradrenaline lead to vasoconstriction, reducing blood flow to the fetus and placenta, potentially causing growth delays (Roescher et al., 2014). Other epigenetic alterations linked to early stress include hypomethylation (or hypermethylation) of the *BDNF* gene (Brain-Derived Neurotrophic Factor), which encodes a neurotrophin critical for brain plasticity. Measuring BDNF levels in the blood and the decrease caused by its gene’s methylation could serve as a marker of behavioral vulnerability induced by environmental stress (Kundakovic et al., 2015).

Stress in early childhood can, over time and as a form of “compensatory adaptation,” lead to reduced reactivity of the corticotropic axis, and long-lasting hypermethylation (persisting 8–16 years) of the *SLC6A4* gene, which encodes the serotonin transporter (SERT). Variants of this gene have been linked to either resilience or hypersensitivity to stress. These changes have been associated with impaired mood regulation and hyperactivity in young children (Timothy et al., 2019).

Among neuropeptides and neurohormones, oxytocin plays a key role. It regulates labor and breast feeding and promotes bonding between mother and child. However, cortisol and sympathetic system overactivation inhibit oxytocin release by the pituitary gland (Walter et al., 2021).

## 6 Attachment and psychosocial bonds—role of the polyvagal system

**Figure 5:**
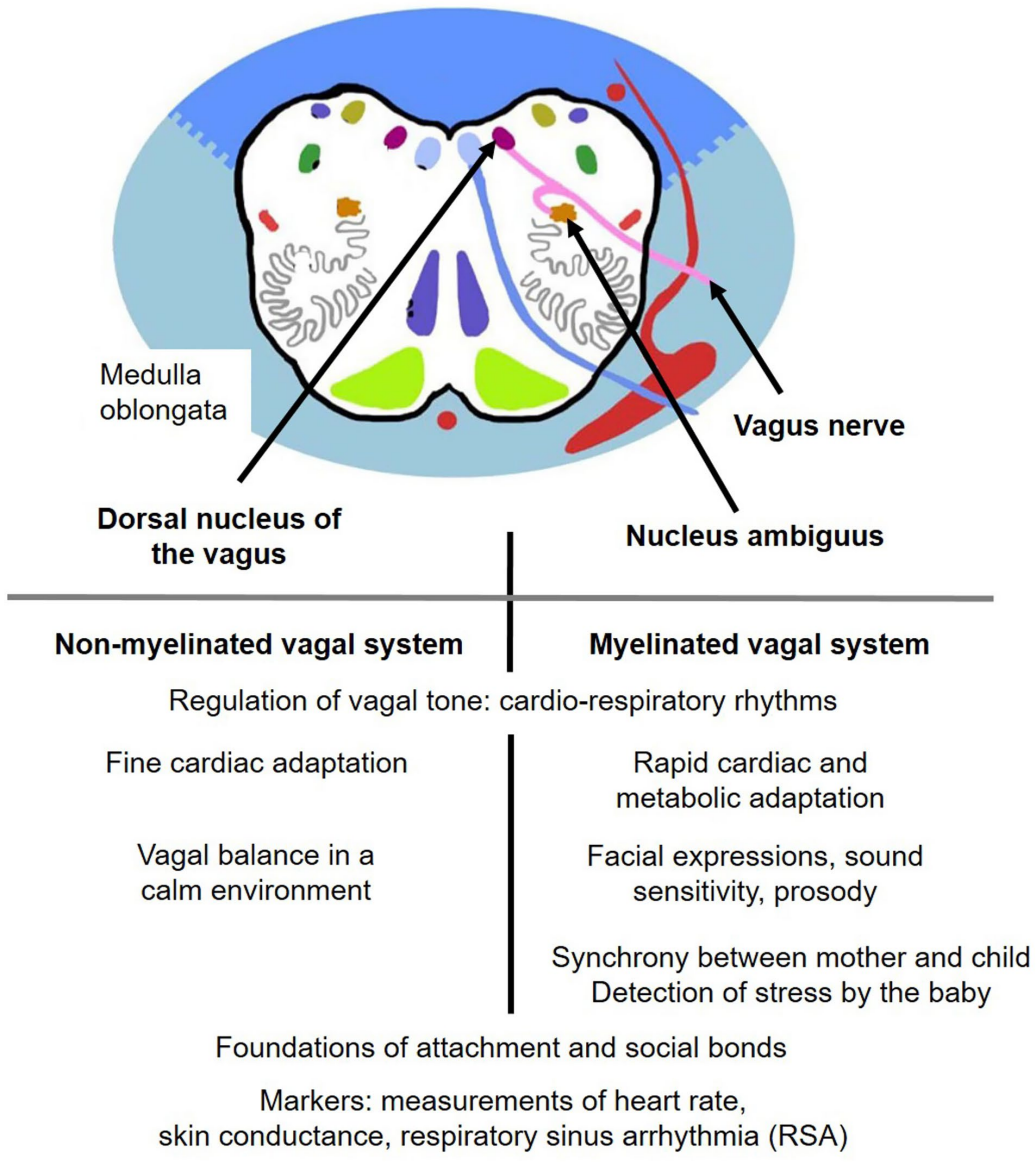
Frontal section of the medulla oblongata illustrating the polyvagal system and its various functions (figure adapted from Neuroclub, University of Geneva). Respiratory sinus arrhythmia (RSA) is heart rate variability in synchrony with respiration, by which the R-R interval on an ECG is shortened during inspiration and prolonged during expiration.

Since the pioneering work of Bowlby (1969) huge progress has been made in early childhood care (Mallinckrodt and Wei, 2005), regarding babies’ early psychosocial skills and their vulnerability to emotional stress (Ilyka et al., 2021; Kingston et al., 2012). The newborn develops their neuroception, their social interaction, associating emotions as joy, anger, or fear (Porges, 2011) (Box 2). Neuroception is defined as automatic and subconscious process by which our nervous system constantly scans the environment and social interactions. The newborn learns by imitation/ synchronization with their caregiver. A caregiver perceived as non-threatening and trustworthy does not trigger reactions from the autonomic and limbic systems and leads to appropriate social interactions. The polyvagal theory introduces the concept of vagal homeostasis of the body’s organs and early psychosocial bonds, beginning at the end of gestation and around birth (Porges, 2022). As explained in Box 2 and Figure 5, the myelinated vagal system is involved in early physical and rhythmic harmonization in babies, mirroring interactions with their caregivers—such as facial expression, heightened auditory sensitivity, and prosody.

Vagal synchrony between mother and child can be assessed through skin conductance, heart rate, and respiratory sinus arrhythmia (RSA) (Figure 5). RSA is used as an index of cardiac vagal function, a physiologic phenomenon reflecting respiratory-circulatory interactions (Busuito et al., 2019). From birth, some newborns display strong temperaments, with many expressive facial gestures in response to positive stimuli from caregivers. Others may appear less expressive (Field et al., 2008). Expressive newborns show higher RSA, which differs from the patterns observed in less expressive infants. Anxious, depressed, or angry mothers tend to have low vagal activity, along with elevated cortisol levels and low levels of dopamine and serotonin. Positive or negative behavioral synchrony between newborns and their mothers has been categorized by Thomas Brazelton into three types of facial expression: happy, sad, or surprised (Tronick et al., 1978). These synchronies are extremely rapid and reversible, as demonstrated by the “Still Face” experiment. For newborns, attachment linked to mirroring interactions with their attachment figures (Thompson et al., 2022). Therefore, the caregiver’s emotional regulation (parent or otherwise) directly initiates harmonious attachment synchrony in the baby. In cases of toxic stress in the baby’s environment, quantifiable clinical evaluations are essential to establish a diagnosis/prognosis and to act as early as possible to protect the infant.

## 7 Discussion: how to support stress adaptation in the vulnerable and developing baby during the first 1,000 days

The risk of exposure to severe stress during early childhood remains high, regardless of geographic context, and may reach a prevalence of up to 50% in neighborhoods affected by violence and crime (Brady et al., 2022; Kaiser et al., 2018). In such environments, some children exhibit cortico-limbic alterations detectable through neuroimaging, in some cases even during the prenatal period. Adversity may arise from abuse, domestic or environmental violence, as well as biological risks such as malnutrition, toxic exposures, or increased susceptibility to infections (Ramaswamy et al., 2021). Therefore, implementing preventive public health policies—particularly those supporting early parenting—is essential to avoid long-term dysregulation of the infant’s physiological and neurodevelopmental systems (Aita et al., 2021). Many newborns can accommodate stress as tolerable, and develop resilience, sometimes resulting in increased motivation to face challenges later in life. More vulnerable infants may experience trauma, leading to emotional dysregulation and adverse health outcomes.

Very premature infants admitted to neonatal intensive care units (NICUs) are exposed to high levels of environmental stress. The Integrated Neonatal Care Model (INC) is defined by seven essential measures for neuroprotective and family-centered care of preterm infants (Altimier and Phillips, 2016). It is a framework that guides clinical practice in many NICUs: (1) a healing environment, (2) partnership with families, (3) positioning and handling, (4) sleep protection, (5) stress and pain reduction, (6) skin protection, and (7) nutrition optimization.

Maternal separation disrupts the infant’s vagal self-regulation and interferes with the synchrony necessary for the development of secure attachment and early social bonding. Nevertheless, premature infants, like all newborns, possess innate adaptive capacities (Gonzalez-Gomez et al., 2021). Some neonatal units now incorporate “family rooms,” allowing parents to remain close to their infant. Parental engagement, including reading aloud in the NICU, may improve physiological stability and support later language development in preterm infants (Scala et al., 2018; Neri et al., 2021).

The “kangaroo method,” first implemented in Bogotá, Colombia, more than 35 years ago as a response to insufficient resources for the care of extremely premature infants, provides an effective strategy to mitigate maternal separation (Pathak et al., 2023). This skin-to-skin contact approach has demonstrated substantial benefits, including improved cardiorespiratory stability, thermal regulation, sleep organization, and strengthened parent–infant attachment. It is also associated with reduced activation of the HPA axis and increased oxytocin release in both the infant and caregiver (Moberg et al., 2020; Pathak et al., 2023). Elevated oxytocin levels in the mother, interacting with dopaminergic reward pathways, enhance positive affect and caregiving motivation. The infant’s sense of safety and wellbeing emerges from the perception of a calm and regulated internal state, mediated by myelinated vagal pathways (Figure 5), and supported through synchronized interactions with caregivers (Porges, 2022).

Premature infants are also more frequently delivered by cesarean section, which can influence the establishment of the gut microbiome (Lammertink et al., 2021; Indrio et al., 2017). While vaginal birth facilitates microbial transfer from the mother, cesarean delivery results in colonization by environmental microbes. The gut microbiome plays a critical role in neonatal homeostasis, and breastfeeding is strongly recommended as a key factor supporting optimal neurodevelopment and immune function (Nelson and Gabard-Durnam, 2020).

**Figure 6:**
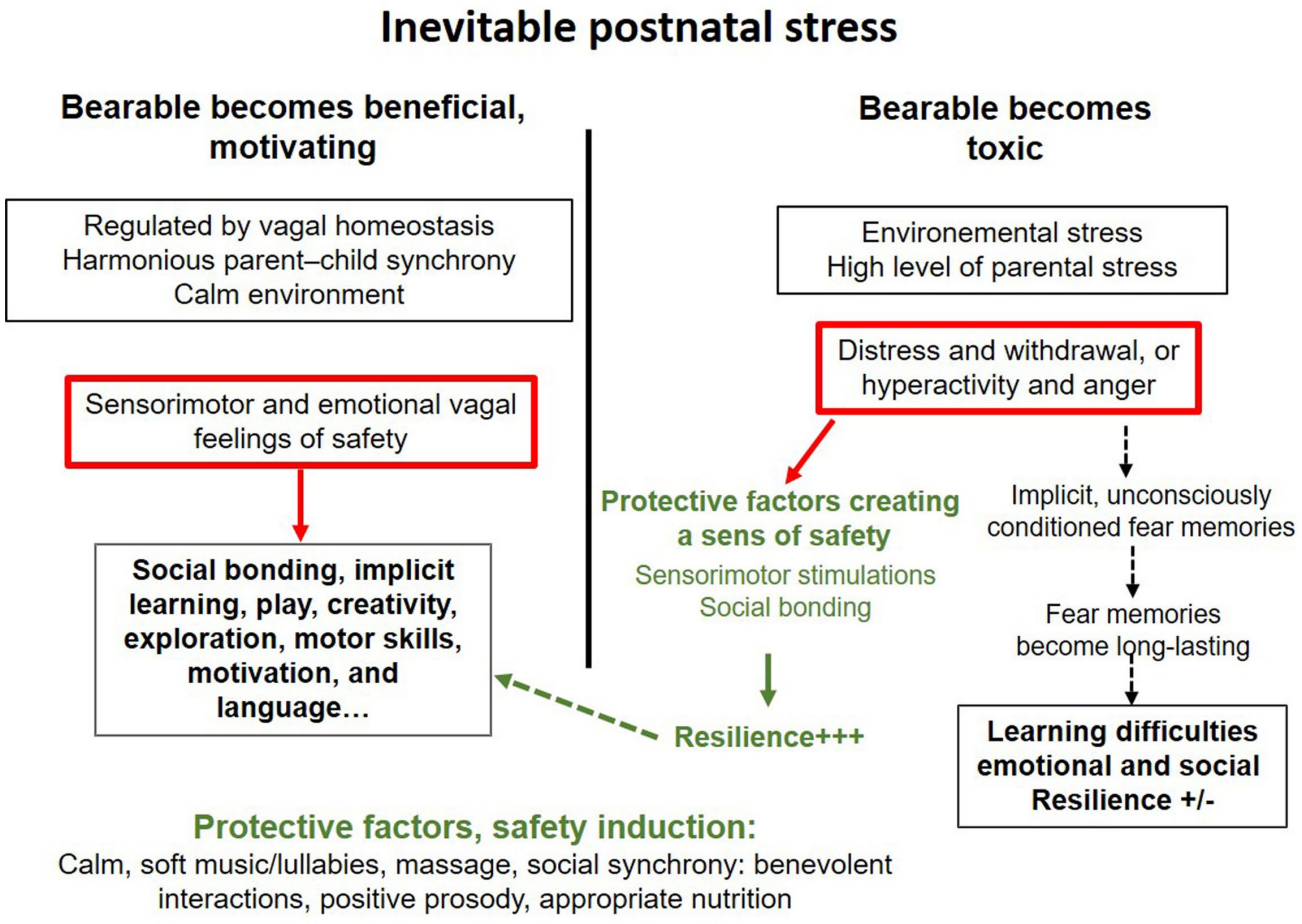
Excessive stress, not manageable by the individual cerebral and emotional plasticities of each neonate, establishes fear, separation anxiety, and a sense of distress (Porges, 2022). In an insecure environment, the neonate either stops exploring and becomes mute or expresses distress through crying, anger, and excessive hyperactivity, with all the possible variations between these two states. These behaviors, observed by caregivers and/or assessed in specialized hospital units (Lammertink et al., 2021) signal the baby’s suffering and guide interventions toward safety, as well as protective factors and support for parenting (Les 1000 premiers jours, 2020).

In everyday life, occasional stress—often interoceptive (e.g., digestive discomfort) but also environmental—is inevitable and may temporarily disrupt caregiver–infant interactions (Figure 6). Soothing practices such as rocking, positive facial and vocal engagement, infant massage, and exposure to gentle music can help restore a state of calm and safety. These interactions support limbic regulations facilitating early implicit learning and the development of foundational executive functions through sensorimotor and attentional processes.

## 8 Conclusion

Early-life stress including prenatal adversity, maternal anxiety, or environmental challenges can alter the buildout of neural networks, particularly in the amygdala, hippocampus and salience network. Fetal and neonatal studies indicate changes in hypothalamic–pituitary–adrenal axis activity, reduced vagal tone, and early functional connectivity disruptions. Epigenetic modifications in genes (*NR3C1, FKBP5, BDNF*) have been detected in infants exposed to maternal stress, linking early adversity to altered stress reactivity and emotional regulation. Functional MRI and physiological assessments indicate that these alterations are detectable before birth and during the first 1,000 days of life, highlighting the high sensitivity of early time windows. Early care strategies produce measurable biological effects as skin-to-skin contact reduces cortisol levels, increases vagal activity and oxytocin release. These interventions directly influence the buildout of neural circuits, hormonal regulation, and epigenetic pathways. Such intermediation emphasizes the concern of monitoring and supporting stress regulation in early life to optimize further neurodevelopmental outcomes.
